# A predictive pharmacokinetic–pharmacodynamic model of tumor growth kinetics in xenograft mice after administration of anticancer agents given in combination

**DOI:** 10.1007/s00280-013-2208-8

**Published:** 2013-06-29

**Authors:** Nadia Terranova, Massimiliano Germani, Francesca Del Bene, Paolo Magni

**Affiliations:** 1 Dipartimento di Ingegneria Industriale e dell’Informazione, Università degli Studi di Pavia, Via Ferrata 3, 27100 Pavia, Italy; 2 PK & Modeling, Accelera srl, V.le Pasteur 10, 20014 Nerviano, MI Italy

**Keywords:** Pharmacokinetic–pharmacodynamic model, Tumor growth inhibition model, Drug combination therapy, Drug interaction, Xenograft mice

## Abstract

**Purpose:**

In clinical oncology, combination treatments are widely used and increasingly preferred over single drug administrations. A better characterization of the interaction between drug effects and the selection of synergistic combinations represent an open challenge in drug development process. To this aim, preclinical studies are routinely performed, even if they are only qualitatively analyzed due to the lack of generally applicable mathematical models.

**Methods:**

This paper presents a new pharmacokinetic–pharmacodynamic model that, starting from the well-known single agent *Simeoni TGI model*, is able to describe tumor growth in xenograft mice after the co-administration of two anticancer agents. Due to the drug action, tumor cells are divided in two groups: damaged and not damaged ones. The damaging rate has two terms proportional to drug concentrations (as in the single drug administration model) and one interaction term proportional to their product. Six of the eight pharmacodynamic parameters assume the same value as in the corresponding single drug models. Only one parameter summarizes the interaction, and it can be used to compute two important indexes that are a clear way to score the synergistic/antagonistic interaction among drug effects.

**Results:**

The model was successfully applied to four new compounds co-administered with four drugs already available on the market for the treatment of three different tumor cell lines. It also provided reliable predictions of different combination regimens in which the same drugs were administered at different doses/schedules.

**Conclusions:**

A good and quantitative measurement of the intensity and nature of interaction between drug effects, as well as the capability to correctly predict new combination arms, suggest the use of this generally applicable model for supporting the experiment optimal design and the prioritization of different therapies.

**Electronic supplementary material:**

The online version of this article (doi:10.1007/s00280-013-2208-8) contains supplementary material, which is available to authorized users.

## Introduction

The use of combination therapies (administration of two or more different drugs) has become a widely adopted strategy in the treatment of patients with cancer, thanks to its advantages over single agent administrations [5]. Drug cocktails, in fact, can often provide a more flexible treatment, characterized by a better response with reduced toxicological effects also thanks to the possibility of attacking the tumor at the same time through different biological pathways.

Therefore, many pharmacompanies and researchers have focused their interest and put several efforts to discover effective drug combinations [23, 24, 28, 29]. In particular, the evaluation of the most promising combinations of a new compound with other anticancer agents, including those already available on the market, is become a fundamental step in early drug development for obtaining a complete description of the compound characteristics [20]. For this purpose, ad-hoc in vitro and in vivo experiments, based on cell cultures and tumor-bearing animals, are routinely performed to assess if the combination has a synergistic, additive or antagonistic interaction (i.e., the effect of the combination is more/equal/less what would be as predicted from the knowledge of the monotherapies) [2, 7, 8, 11, 16]. One of the main objectives of screening experiments during the early drug development is to identify drug combinations that yield an enhanced pharmacological effect and to prioritize them according to their interaction intensity. During this phase, many variations of drug combinations are tested, but each drug combination might be tested only at one or two dose levels [14]. Although these experiments certainly contribute to a better description of the activities of a new compound, their quantitative interpretation and the abstraction of general valid conclusions from the specific experimental design are not trivial tasks. For example, even if there are several approaches to evaluate in vitro data (e.g., [4, 7]), these methods strongly depend on the experimental settings and they are not suitable for comparing experiments with different designs [12]. The choice of endpoints is crucial as well. The task becomes more complex when the evaluation has to be done in vivo, especially because it is confounded by the dynamics of the tumor growth (present also in non-treated animals) and the above-mentioned approaches are only applicable to endpoints and not to time-course data.

This paper is focused on in vivo studies. As reported in [25], xenograft models are, in the oncology therapeutic area, the most popular preclinical models for evaluating the anticancer activity of compounds still under development [15]. Despite some limitations, their implementation is relatively easy and requires limited resources. Traditional approaches to the measurement of efficacy have been based on the ratio of the tumor volumes/weights in treated and control animals at a specific endpoint. Unfortunately, most of these tumor growth inhibition metrics are not invariant with respect to the experimental conditions. For instance, the time to maximal value of the tumor growth inhibition was shown to be dose-, time- and dosing regimen-dependent [21]. Only mathematical models that are able to describe tumor growth by dissecting the system-specific properties can provide compound-specific and experiment-independent model parameters. For this reason, several mathematical models have been proposed, in the last decade, to describe the relationship between the drug pharmacokinetics (PK) and suppression of tumor growth rates explicitly considering the time variable (see the reviews [3, 6, 25]). These models are capable of predicting the antitumor effect of a single compound as a function of the dosing regimen. They are also indicated as possible suitable tools for extracting the descriptors of these processes, which can be translated from preclinical to clinical setting [22, 25].

Among these models, the *Simeoni tumor growth inhibition (TGI) model* [18, 26] is the most popular and, often, acts as a reference. It has been successfully used for analyzing hundreds of single agent experiments and even several general purpose or specialized software tools included it in their model library [1, 9, 27].

The *Simeoni TGI model* has also been modified to cope with combination therapies to provide an in vivo evaluation of the interaction between drug effects [10, 14]. However, as better discussed in the conclusions, these models suffer from some limitations. An interesting and a completely different approach is that adopted in [19, 20]. Combining two or more single drug TGI models and applying the Bliss independence criterion [11] in a dynamic context, a no interaction (or zero-interaction) model was proposed. In particular, instead of fitting a drug-drug interaction model, the simulations obtained by the zero-interaction model are compared with the experimental data of the combination therapy; the validity of zero-interaction hypothesis is then assessed by a suitable statistical test. Therefore, the model is able to predict the tumor growth inhibition in case of the “additivity” of the combined drugs effects, whereas the synergistic or antagonistic behaviors are derived from departures of the experimental data from the predictions. Under the assumption of a pharmacodynamic (PD) null interaction, the parameters relative to the drug potencies preserve in combination the same values of those derived in single agent regimens.

Despite these recent attempts, as also reported in [10, 25], it is a current opinion that one of the major gaps in the preclinical characterization of new anticancer compounds is the lack of a general combination model able to predict the inhibition of the tumor growth curve in response to interacting drugs given in combination. The model has to be simple enough to be identified on experimental data available during the preclinical phase, in which, as already highlighted, many drug combinations are tested, but each drug combination might be tested only at one or two dose levels.

Then, starting from the *Simeoni TGI model* and considering the specific mentioned constraints, in this paper, we propose and extensively test a new general model able to fill this gap. It describes and predicts the inhibition of the tumor growth curve in response to two co-administered drugs, and it allows to assess the nature and the intensity of the interaction in any considered combination experiment.

## Materials and methods

### Experimental methods

#### Compounds

Four novel anticancer compounds still under development (hereafter called Drug C1, Drug C2, Drug C4, Drug C5 1), synthesized by Nerviano Medical Sciences, Nerviano, have been tested (after a preliminary evaluation in vitro) in xenograft mice in combination with the following drugs already used in the standard clinical treatment of specific tumors: Irinotecan (CPT-11), 5-fluorouracil (5-FU), Cisplatin and Gemcytabine. In total, six experiments, testing 11 different combination treatments involving more then 230 mice, were led. Three of them were partially analyzed in [20], where they are indicated as experiment 1, 2 and 3. The other three experiments, instead, are unpublished data coming from combination therapies that involve Drug C4, Drug C5 and the Drug C1 administered in a new combination treatment.

#### Animals, cell lines and in vivo tumor growth experiments

Female CD1 athymic nude-nu mice, 5 weeks of age (17–35 g), used in the combination experiment relative to Drug C1 and Gemcytabine (*Experiment a*, see the following subsection) and male Balb, athymic nude-nu mice, 5 weeks of age (25−35 g) used in all the other experiments, were obtained from Harlan, S. Pietro al Natisone, Italy.

A2780 human ovarian carcinoma, HT29 human colon adenocarcinoma and BxPC3 human pancreatic adenocarcinoma cell lines (from American Type Culture Collection) were maintained by s.c. transplantation in athymic mice using 20–30 mg of tumor brei. Tumors were excised and fragments were implanted s.c. into the left flank. One week after inoculation, mice bearing a palpable tumor (approximately 100−300 mm^3^) were randomized into control and treatment groups (usually eight animals for group). Then, mice were treated with anticancer compounds and clinically evaluated daily. Dimension of the tumors were measured, usually every two or three days, using callipers, and tumor masses (mg) were calculated as length (mm)·width^2^ (mm^2^)/2, assuming unit density.

All of the experiments were conducted in accordance with the current best practices and ethic principles.

#### Drug treatments

Drug C1 was tested in combination with Gemcytabine (*Experiment a*) and Cisplatin (*Experiment b*). In particular, Drug C1 was given to mice bearing BxPC3 tumor cells at 15 mg/kg (29.4 μM/kg) i.v. two times per day (bid) for three days for three cycles starting from days 10, 14 and 18 in combination with Gemcytabine 80 mg/kg (267 μM/kg) i.v. every four days for three times (q4dx3) starting from day 9 2. The second experiment has two combination arms. Drug C1, 30 mg/kg (58.7 μM/kg) i.v. bid for 5 days starting from day 8 in the first combination arm and from day 9 in the second one, was administered to mice bearing A2780 tumor cells in combination with Cisplatin given i.v. at 8 mg/kg (26.7 μM/kg) as single dose at day 13 and 8 in the first and the second combination arms, respectively.

Drug C2 was orally given on days 10, 11, 12, 14, 15 and 16 at 45 mg/kg (84.5 μM/kg) and 60 mg/kg (113 μM/kg) in combination with CPT-11, given i.v. q4dx3 starting from day 9 at 45 mg/kg (72.9 μM/kg) (*Experiment c*). Drug C2 was also administered in combination with 5-FU (*Experiment d*) at 50 mg/kg (384 μM/kg) following the same schedule and at the same dose level (for Drug C2) of the *Experiment c*. In both experiments, HT29 tumor-bearing mice have been treated.

Drug C4 was orally administered to mice bearing BxPC3 tumor cells bid for three days for three cycles starting from day 9 or 10 3, 14 and 18 at 20 mg/kg (37.5 μM/kg) and 40 mg/kg (75 μM/kg) in two different combination arms with Gemcitabine 80 mg/kg (267 μM/kg) i.v. q4dx3 starting from day 9 (*Experiment e*).

Drug C5 was administered in combination with CPT-11 to HT29 tumor-bearing mice. Drug C5, i.v daily administered for 8 days from day 9 at two different dosages (10 mg/kg, 25.1 μM/kg and 20 mg/kg, 50.3 μM/kg), was given in combination with CPT-11, i.v. q4dx3 starting from day 8 at 45 mg/kg (72.9 μM/kg) (*Experiment f*). In the administration days in which both compounds had to be administered, Drug C5 was actually given 6 h later then CPT-11.

In each of the six considered combination experiments, three additional arms are present: a control arm in which mice were not treated with any drug and two arms in which the anticancer agents were given separately following the same schedule of the corresponding combination.

Table 1 summarizes the combination treatments part of this study.

**Table 1:** Explored combination treatments

	Cell line	Arm	Compounds	Days of administration in the combination arms
*Experiment a*	BxPC3	*a*_1_	Drug C1 (15 mg/kg)	10, 10.5, 11, 11.5, 12, 12.5, 14, 14.5, 15, 15.5, 16, 16.5, 18, 18.5, 19, 19.5, 20, 20.5
			Gemcytabine (80 mg/kg)	9, 13, 17
*Experiment b*	A2780	*b*_1_	Drug C1 (30 mg/kg)	8, 8.5, 9, 9.5, 10, 10.5, 11, 11.5, 12, 12.5
			Cisplatin (8 mg/kg)	13
		*b*_2_	Drug C1 (30 mg/kg)	9, 9.5, 10, 10.5, 11, 11.5, 12, 12.5, 13, 13.5
			Cisplatin (8 mg/kg)	8
*Experiment c*	HT29	*c*_1_	Drug C2 (45 mg/kg)	10, 11, 12, 14, 15, 16
			CPT-11 (45 mg/kg)	9, 13, 17
		*c*_2_	Drug C2 (60 mg/kg)	10, 11, 12, 14, 15, 16
			CPT-11 (45 mg/kg)	9, 13, 17
*Experiment d*	HT29	*d*_1_	Drug C2 (45 mg/kg)	10, 11, 12, 14, 15, 16
			5-FU (50 mg/kg)	9, 13, 17
		*d*_2_	Drug C2 (60 mg/kg)	10, 11, 12, 14, 15, 16
			5-FU (50 mg/kg)	9, 13, 17
*Experiment e*	BxPC3	*e*_1_	Drug C4 (20 mg/kg)	10, 10.5, 11, 11.5, 12, 12.5, 14, 14.5, 15, 15.5, 16, 16.5, 18, 18.5, 19, 19.5, 20, 20.5
			Gemcytabine (80 mg/kg)	9, 13, 17
		*e*_2_	Drug C4 (40 mg/kg)	10, 10.5, 11, 11.5, 12, 12.5, 14, 14.5, 15, 15.5, 16, 16.5, 18, 18.5, 19, 19.5, 20, 20.5
			Gemcytabine (80 mg/kg)	9, 13, 17
*Experiment f*	HT29	*f*_1_	Drug C5 (10 mg/kg)	9, 10, 11, 12, 13, 14, 15, 16
			CPT-11 (45 mg/kg)	8, 12, 16
		*f*_2_	Drug C5 (20 mg/kg)	9, 10, 11, 12, 13, 14, 15, 16
			CPT-11 (45 mg/kg)	8, 12, 16

#### Pharmacokinetic studies

Since the PK of CPT-11, 5-FU, Cisplatin, Gemcytabine, Drug C1 and Drug C2 were already assessed in [20, 22], no new PK studies were performed as part of this work. Conversely, the PK of the candidates Drug C4 and Drug C5 were investigated in separate groups of mice. Blood samples were collected, and drugs were assayed in plasma by LC–MS–MS, following a standard procedure generally adopted in the drug discovery phase [13].

### Mathematical modeling

#### Pharmacokinetic model

All the drugs involved in this study showed a linear PK well described by a standard one/two compartment model, with a first-order absorption in case of oral administration.

#### Pharmacodynamic model

The development of a new predictive model able to describe the time course of the tumor growth under combination regimens started from the models mentioned in the introduction and, in particular, from the *Simeoni TGI model*.

The control tumor growth curve (untreated animals) was described by the same equation originally proposed in [26] being not affected by the drug administration and, then, it is characterized by two parameters, originally called λ_0_ and λ_1_. For what concerns treated animals, we still suppose that drugs damage tumor cells that become non-proliferating and eventually die after having passed a certain number of damage states. Then, the drug action is characterized by two parameters, originally called *k*_2_ (the drug potency) and *k*_1_ (linked to the death delay) [26]. In presence of therapies in which drugs are given in combination, we suppose that no interaction affects the PK. This hypothesis can be immediately removed if an appropriate PK study highlights a PK interaction effect. In addition, we assume that once a cell is hit by a drug can still be hit also from the other drug. Therefore, if in the single agent *Simeoni TGI model* there are 4 states in which a cell can be (1 state for not damaged cells and 3 states for damaged ones), in the new model 16 (4 × 4) states have to be defined (1 state for not damaged cells, 3 states for cells damaged only by drug A, 3 states for cells damaged only by drugs B and 3 × 3 states for cells damaged by both drugs). Starting from these assumptions, some hypotheses were formalized, introduced into a mathematical model and tested.

**Fig. 1:**
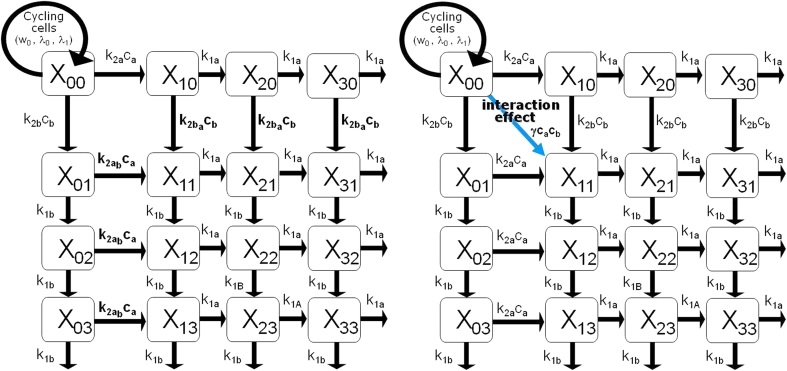
Schematic representation of the TGI model for two drugs given in combination: a first attempt (*left panel*) and the final proposed model (*right panel*)

In the first attempt, we assumed that the killing potency of a drug on the tumor cells already damaged by another drug could be modified by the simultaneous action of the second drug increasing it in case of synergism or decreasing it in case of antagonism. This hypothesis leads to the introduction of two additional parameters $k_{2a_b}$ and $k_{2b_a}$in the TGI model formulated for two drugs, describing the drug potency of compound *a* on the cells already damaged by drug *b* and the drug potency of compound *b* on the cells already damaged by drug *a*, respectively (Fig. 1, left panel).

Then, the comparison of these potency parameters with those of the single agent regimens (*k*_2*a*_ and *k*_2*b*_) could provide a measurement of PD interaction. Nevertheless, this first attempt showed identification problems that were investigated by performing a simulation analysis. It was possible to observe that the simulated tumor growth curves in combination regimen did not significantly change one from the other, even considerably varying the potency parameters $k_{2a_b}\,\hbox{and}\,k_{2b_a}.$ In fact, since the introduced parameters influence only the non-proliferating cells that in the typical setting constitute only a small part of the total tumor mass, the values of $k_{2a_b}\,\hbox{and}\,k_{2b_a}$ do not relevantly impact on the growth curve time course. Similar considerations can be addressed for a possible variation of the *k*_1_ parameters due to the interaction between the drug effects. Therefore, an interaction term had to be introduced on the proliferating cell compartment. This term was assumed to be proportional to the weight of the proliferating cells and to the drug concentrations through a parameter γ (the interaction parameter). Thus, the final proposed model (Fig. 1, right panel) can be formulated through the following differential equations:

1 $$ \dot{x}_{00}(t) = f_p(w(t)) - (k_{2a}c_a(t)+k_{2b}c_b(t) +v_{11})x_{00} $$

2 $$ \dot{x}_{ij}(t) = u_{aij}+u_{bij} +v_{ij} \quad \quad \quad i+j>0 $$

3 $$ \begin{aligned} w(t) & = \sum_{i=0}^{3}\sum_{j=0}^{3}x_{ij}(t) \\ x_{00}(0) &= w_0, \quad x_{ij}(0)= 0 \quad i+j>0 \end{aligned} $$

where

$$ \begin{aligned} f_p(w(t)) & = \frac{\lambda_{0}x_{00}(t)}{\left[ 1+\left(\frac{\lambda_{0}}{\lambda_{1}}w(t)\right) ^{\Uppsi}\right]^{\frac{1}{\Uppsi}}}\\ u_{aij} & = \left\{ \begin{array}{ll} 0 & i=0\\ k_{2a}c_a(t)x_{i-1,j} - k_{1a}x_{ij} & i=1\\ k_{1a}x_{i-1,j} - k_{1a}x_{ij}& i=2,3 \end{array}\right.\\ u_{bij} & = \left\{\begin{array}{ll} 0 & j=0\\ k_{2b}c_b(t)x_{i,j-1} - k_{1b}x_{ij} & j=1\\ k_{1b}x_{i,j-1} - k_{1b}x_{ij}& j=2,3 \end{array}\right.\\ v_{ij} & = \left\{\begin{array}{ll} \gamma c_a(t) c_b(t)x_{00} & i=j=1 \\ 0 & \hbox{otherwise} \end{array}\right. \end{aligned} $$

Considering that the potency of each drug could change due to the interaction between drug effects, the interaction term (*v*_11_) cannot be disregarded as in the case of no interacting (or additive) effects, but it can be positively or negatively modulated by the parameter γ. If the value of γ is higher than, lower than or close to zero, the interaction of drug effects has a synergistic, antagonistic or additive nature, respectively. It is important to note that the two additional parameters $k_{2a_b}\,\hbox{and}\,k_{2b_a}$, introduced in the first attempt, were not included in the final version of the model (Fig. 1) because, as discussed before, only the effect interaction on the proliferating cells can be significantly appreciated on the tumor mass dynamics. Last but not least, this model in such formulation, as shown in the "Results" section, can be easily identified and well describes the experimental data at the same time.

Characterized the nature of the PD interaction in one combination experiment through the parameter γ, it is of interest to compare results of different experiments in order to select the most promising combinations, in terms of strengthened antitumor effects. Nevertheless, the parameter γ cannot be used directly to compare different combination treatments or to rank them in accordance to it, because its value is not a pure measure of strength of the drug effect interaction but it depends from the potencies of the considered drugs. Conversely, the evaluation of the horizontal distance between the predictive tumor growth curve (PTGC), obtained by the zero-interaction model under the hypothesis of no interaction between drug effects [20], and the curve obtained by the new combination TGI model can provide a very useful index to quantify the contribute on the TGI imputable to the drug effect interaction.

To this aim, the *Time Efficacy Index* (TEI) [18] for combination treatments and two new related indexes were defined. TEI is an antitumor efficacy measure already defined and largely used for the single agent TGI model. It is defined as the asymptotic delay between the growth curves of treated and untreated groups. Following the same rationale in [18], the TEI for combination regimens (*TEI*_comb_) has been evaluated and it results well approximated by (see the Supplementary file S1 for more details)

4 $$ TEI_{\rm{comb}} \simeq \frac{k_{2a}AUC_{c_a}+k_{2b}AUC_{c_b}+ \gamma AUC_{c_ac_b}}{\lambda_0} $$

where *AUC*_*x*_ is the area under the curve *x*.

The *TEI*_comb_ index measures the overall antitumor efficacy of a combination regimen treatment and can be used to compare competing antitumor drug combinations. However, in order to derive the specific portion of *TEI*_comb_ due to the PD interaction, $\Updelta$ was defined as the time-shift of the growth curve of treated group in combination regimen with respect the PTGC obtained with the zero-interaction model. In the Supplementary file S2, it is shown that

5 $$ \Updelta \simeq \frac{\gamma AUC_{c_ac_b}}{\lambda_0} $$

Finally, two very intuitive normalized indexes, always in the range 0–100, were defined to better express the percentage contribution of the PD interaction: one for synergistic combinations and one for antagonistic ones. In particular, the *synergistic combination index* (*SC*) can be defined for $\Updelta >0$ as

6 $$ SC \equiv 100 \cdot \frac{\Updelta }{{TEI_{{{\text{comb}}}} }} $$

whereas the *antagonistic combination index* (*AC*) can be defined for $\Updelta <0$ as

7 $$ AC \equiv 100 \cdot \frac{-\Updelta}{TEI_{\text{add}}} $$

where *TEI*_add_ is the theoretical TEI computed considering the PTGC obtained with the zero-interaction model.

### Data analysis

PK and PD models were implemented in WinNolin (version 3.1, Pharsight, Mountain View, CA) and MATLAB (version 2007b, The MathWorks, Inc.). The concentration profiles required by the TGI model were simulated for each experiment using the PK parameters estimated in single drug studies. The PK data of Drug C4 and Drug C5, obtained in satellite groups, were averaged and used to identify the kinetic parameters. The tumor growth data were analyzed adopting the following strategy. First, seven PD parameters were estimated by fitting the *Simeoni TGI model* against the average data of control and treated groups considering only single agent arms. That allows to share the same tumor-related parameters *w*_0_, λ_0_ and λ_1_ between groups and, on the other hand, to identify the different drug-related parameters *k*_1*a*_, *k*_2*a*_, *k*_1*b*_, *k*_2*b*_. Then, fixing these parameters to the estimated values, the new proposed TGI model was simultaneously fitted, for each combination, against the control and the combination arms to obtain the value of the interaction term γ. Note that a direct simultaneous fitting of the combination model on all the arms without fixing any model parameter is possible and, perhaps, it can improve in some cases even the parameter estimation. However, the suggested two-step strategy allows to capture all the drug effect interaction in a single parameter that can be subsequently used to compare and rank the different combination therapies without introducing biases due to the simultaneous variation of the monotherapies linked parameters. Moreover, in the drug development process, a number of single drug experiments can be performed in an early phase and then single agent model parameters can be made available before.

Model identification was performed by using the nonlinear weighted least squared algorithm (with weights equal to 1/*y*_observed_^2^) 4. In those cases in which several arms were fitted together, residuals were normalized in order to give the same weight to each arm 5.

## Results

### Pharmacokinetic model

The PK parameters of CPT-11, Gemcytabine, 5-FU, Cisplatin, Drug C1 and Drug C2 were previously derived in [20, 22] and are reported in the Supplementary Table 1. The PK parameters of Drug C4 and Drug C5 were estimated as part of this study from experimental data (data not shown). The estimated values are reported in the Supplementary Table 1 as well.

### TGI model identification

#### Drug C1

**Fig. 2:**
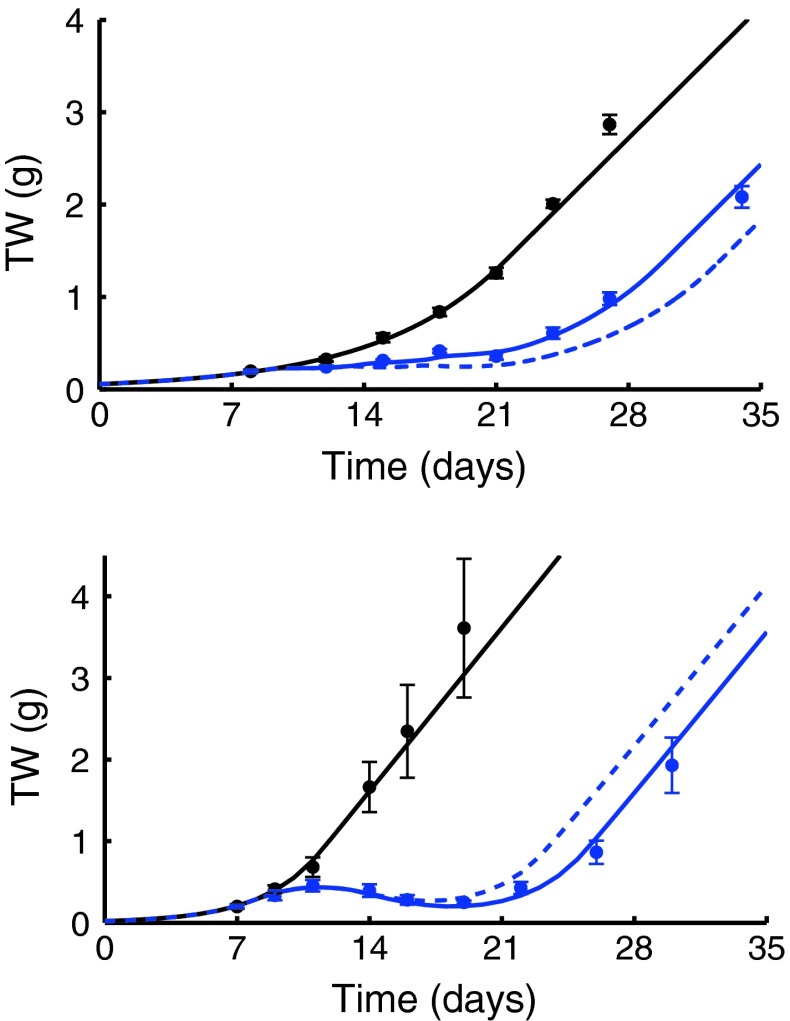
The fitted tumor growth curve (*blue continuous line*) of the combination treatment, the PTGC of the related zero-interaction model (*dashed line*) and the control curve (*black line*) are reported together with the experimental data (*circles*: mean values, *vertical bars*: one standard errors). In the top panel, combination between Drug C1 (15 mg/kg) and Gemcytabine (80 mg/kg); in the bottom panel, first combination between Drug C1 (30 mg/kg) and Cisplatin (8 mg/kg), arm *b*_1_

Considering the first experiment relative to Drug C1 given in combination with Gemcytabine (*Experiment a*), seven PD model parameters (i.e., *w*_0_, λ_0_, λ_1_, *k*_1*a*_, *k*_1*b*_, *k*_2*a*_, *k*_2*b*_) were fixed to the values previously estimated in [20] (Experiment 1) from the control and single agent arms (values are reported in the Supplementary Table 2). The interaction parameter γ of the TGI model was estimated, fitting the combination arm, equal to −0.88 μM^−2^day^−2^ with a coefficient of variation (CV) of 10 %. 6 The experimental data are well described by the model (see Fig. 2, top panel). A root-mean-square error (RMSE) of 0.19 g was computed.

The estimated antagonistic combination index *AC* (24 %) shows a significant role of the negative interaction.

Drug C1 was also studied in combination with Cisplatin at two different doses and schedules (*Experiment b*). Seven PD parameters were preliminary estimated through simultaneous fitting of three arms: the control, single agent Drug C1 (bid from day 9 to day 13) and single agent Cisplatin (single dose at day 8). The estimates and their CVs (between brackets) are reported in the Supplementary Table 2. The RMSE is 0.26 g.

Then, fixing these parameters, the first combination arm (*b*_1_) was fitted (RMSE = 0.17 g) and the interaction parameter was estimated, γ = 8.0 μM^−2^day^−2^ (CV = 15 %). In Fig. 2, bottom panel, the fitted tumor growth curves for the combination and the control arms are shown together with the experimental data. Even if the positive value of γ could suggest a significant synergistic interaction (its absolute value is tenfold of the previous combination study), the computation of the synergistic combination index *SC* = 14.7 % highlights a moderate synergistic effect. Drug C1 shows a different behavior when administered in combination with different drugs on different tumor cell lines.

#### Drug C2

Drug C2 was studied in combination with CPT-11 (*Experiment c*) and 5-FU (*Experiment d*). In both experiments, seven PD parameters were fixed to the values previously estimated in [20] (Experiment 2 and 3, respectively) from the control and single agent arms (values are reported in the Supplementary Table 2). Then, the combination arms were fitted with the proposed TGI model to estimate the combination parameters.

**Fig. 3:**
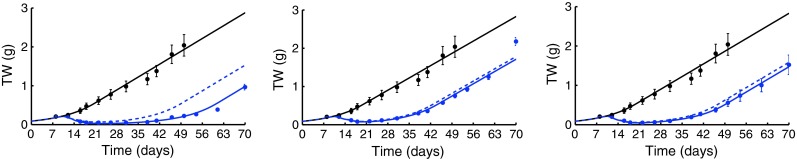
The fitted tumor growth curve (*blue continuous line*) of the combination treatment, the PTGC of the related zero-interaction model (*dashed line*) and the control curve (*black line*) are reported together with the experimental data (*circles*: mean values, *vertical bars*: one standard errors). In the left panel, first combination between Drug C2 (45 mg/kg) and CPT-11 (45 mg/kg), arm *c*_1_; in the central panel, first combination between Drug C2 (45 mg/kg) and 5-FU (50 mg/kg), arm *d*_1_; in the right panel, second combination between Drug C2 (60 mg/kg) and 5-FU (50 mg/kg), arm *d*_2_

In particular, considering the first combination arm of the *Experiment c* the parameter γ was estimated to 3.45 μM^−2^day^−2^ (CV = 11 %). The fitted curve of the combination arm together with the experimental data and the control arm are shown in Fig. 3, left panel. The RMSE is 0.07 g.

The synergistic combination index *SC*, equal to 29 %, suggests an important interaction between the two drugs given in combination. In fact, almost one-third of the total inhibition is due to the interaction effect.

Considering the *Experiment d*, both the combination arms were independently fitted 7 with the proposed TGI model. Results are shown in Fig. 3 central and right panels.

The combination parameter was estimated to very similar values in both arms: γ_1_ = 0.131 μM^−2^day^−2^ (CV = 42 %) for the first combination with RMSE = 0.11 g and γ_2_ = 0.157 μM^−2^day^−2^ (CV = 36 %) for the second combination arm with RMSE = 0.06 g. A very limited synergistic interaction is suggested by the *SC* values equal to 6 and 8 %, respectively, in the two combination arms. It is quite interesting to remark a non-trivial result: a changing of about 33 % in the dose level of Drug C2 does not affect significantly the interaction intensity.

#### Drug C4

Considering the experiment relative to Drug C4 given in combination with Gemcytabine, the seven PD parameters were preliminary estimated from the single agent experimental arms (RMSE = 0.06 g), i.e., control group, group treated with Drug C4 20 mg/kg, group treated with Gemcytabine 80 mg/kg. The estimates and their CVs (between brackets) are reported in the Supplementary Table 2.

**Fig. 4:**
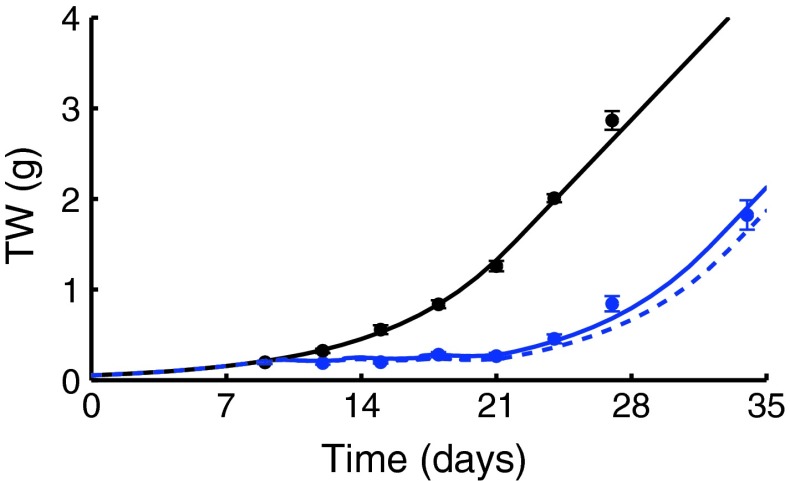
Fitted tumor growth curve of the first combination (*blue continuous line*) between Drug C4 (20 mg/kg) and Gemcytabine (80 mg/kg), arm *e*_1_, the PTGC of the related zero-interaction model (*dashed lined*) and the control curve (*black line*) are reported together with the experimental data (*circles*: mean values, very small *vertical bars*: one standard errors)

Then, the first combination arm, *e*_1_, was fitted (RMSE = 0.09 g) and the combination parameter γ = −0.0455 μM^−2^day^−2^ (CV = 28 %) was estimated. Results are shown in Fig. 4.

The antagonistic combination index *AC* is equal to 10 %, although the small negative values of γ highlights a slight antagonistic interaction effect.

#### Drug C5

The two arms, *f*_1_ and *f*_2_, related to Drug C5 and CPT-11 combination were independently fitted with the model. Seven PD parameters were preliminary estimated (RMSE = 0.19 g) from the control arm, two single agent arms treated with Drug C5 (10 and 20 mg/kg) and a single agent arm treated with CPT-11 45 mg/kg. The estimates and their CVs (between brackets) are reported in the Supplementary Table 2.

**Fig. 5:**
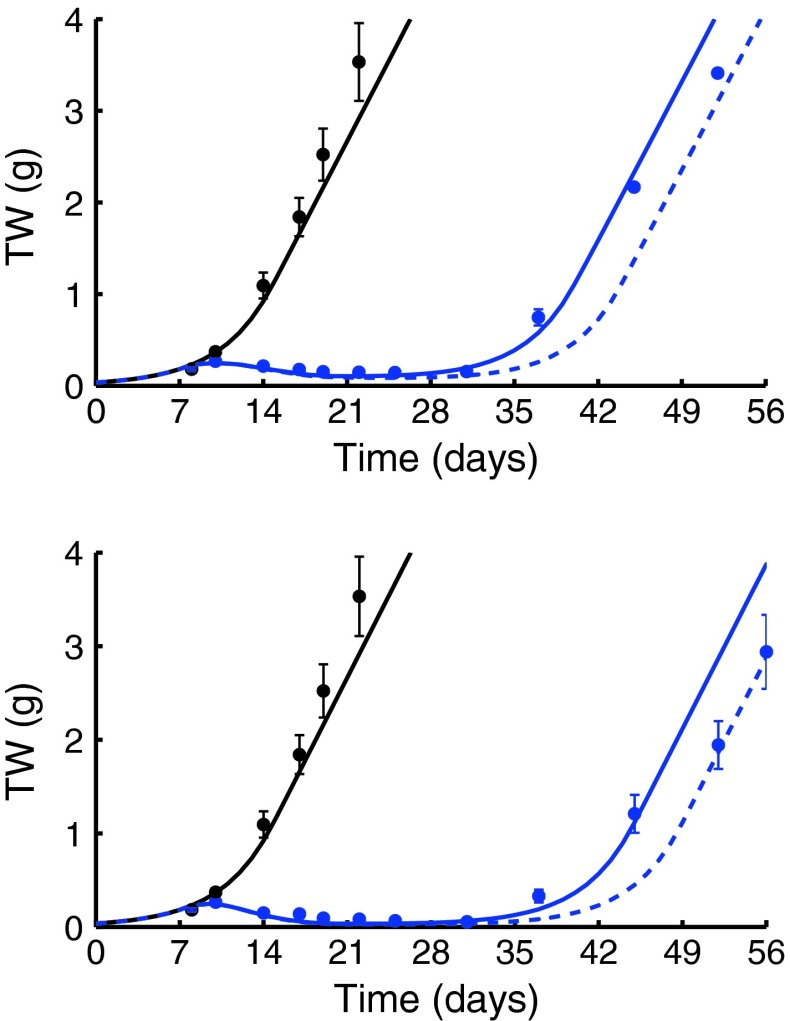
The fitted tumor growth curve (*blue continuous line*) of the combination treatment, the PTGC of the related zero-interaction model (*dashed line*) and the control curve (*black line*) are reported together with the experimental data (*circles*: mean values, *vertical bars*: one standard errors). In the top panel, first combination between Drug C5 (10 mg/kg) and CPT-11 (45 mg/kg), arm *f*_1_; in the bottom panel, the second combination between Drug C5 (20 mg/kg) and CPT-11 (45 mg/kg), arm *f*_2_

Then, the interaction parameters were estimated to negative values for both combination arms: γ_1_ = −2.04 μM^−2^day^−2^ (CV = 18 %) and γ_2_ = −1.05 μM^−2^day^−2^ (CV = 30 %). The fitting showed an RMSE equal to 0.25 and 0.36 g, respectively, for the two different conditions. Results are shown in Fig. 5.

The computed antagonistic combination indexes *AC* = 14 % and *AC* = 12 % indicate a possible antagonistic interaction. Note that, although the two interaction parameters γ are twofold in the two conditions, the combination indexes are very similar.

### TGI model predictive power

Since the proposed TGI model showed excellent fitting capabilities in a number of different situations and similar gamma values in the different arms related to therapies using the same drugs, it was interesting to explore its predictive power. In particular, given the PD parameters estimated in a combination experiment, the behavior of a new combination therapy involving the same drugs administered in a different schedule/dose was predicted and compared with the data actually collected. Three different situations have been explored: a modification of the schedule (Cisplatin is given before or after the administration of Drug C1, i.e., *b*_1_ vs *b*_2_), a 30 % dose increasing (Drug C2 with CPT-11, i.e., *c*_1_ vs *c*_2_) and a 100 % dose increasing (Drug C4 with Gemcytabine, *e*_1_ vs *e*_2_). The administration regimens of the predicted situations are not totally different from those considered in the model fitting step, for the following reasons: (i) generally in case of combination studies each drug is previously widely studied as single agent, therefore the exposures really tested in combination do not cover a wide range but are focused on the single drug results (and only one or two dose levels are considered to avoid a very expensive combinatory approach); (ii) in combination studies, the drug effect interaction is expected to be drug concentration dependent, if a huge range of concentrations is explored, then it is very important to verify the region of validity of the model and discover its limits within a range of reasonable working doses.

**Fig. 6:**
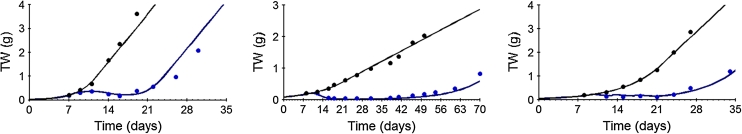
In the left panel, the PTGC curve (*blue line*) of the second combination between Drug C1 (30 mg/kg) and Cisplatin (8 mg/kg), arm *b*_2_, and the control curve (*black line*) are reported together with the experimental data (*circles*: mean values). In the middle panel, the PTGC (*blue line*) of the second combination between Drug C2 (60 mg/kg) and CPT-11 (45 mg/kg), arm *c*_2_ and the control curve (*black line*) are reported together with the experimental data (*circles*: mean values). In the right panel, the PTGC (*blue line*) of the second combination between Drug C4 (40 mg/kg) and Gemcytabine (80 mg/kg), arm *e*_2_, and the control curve (*black line*) are reported together with the experimental data (*circles*: mean values)

The first situation considered the second combination arm of the *Experiment b*, not yet used in the TGI model identification analysis section. In Fig. 6, left panel, the PTGC of the *b*_2_ combination arm obtained by fixing the TGI model parameters to those derived by fitting the control, the single agents and the *b*_1_ combination arms are reported together with the real data.

Note that, no fitting was done in this case on the second arm. Model predictions are quite close to the experimental data (RMSE =  0.32 g), even if the error is greater for the highest tumor weights. Another important measure to assess the TGI predictive power is the comparison of the synergistic/antagonistic combination index predicted by the model identified on another arm with that computed actually fitting the experimental data. In the case of *Experiment b*, the synergistic combination index *SC* predicted by the model is close to zero whereas that computed fitting the data are equal to 8 %. Therefore, also considering this very impacting index, the prediction capability is very good: in both cases, the conclusion is that no significant synergistic neither antagonistic interactions were shown in this combination arm. The result is particular important also taking into account that the schedule in the two arms is completely different (the order of the administration of the two drugs was exchanged, see Table 1).

The second example is the second combination arm of the *Experiment c*. The PTGC of the *c*_2_ arm is reported in Fig. 6, central panel, with the real data. The generated curve very well predicts the experimental data (RMSE = 0.08 g). The synergistic combination index predicted in this case is 32 %, and it suggests a strong interaction between the two drugs. On the other hand, fitting *c*_2_ arm data to obtain the combination parameter γ, the resulting value of the *SC* is 21 % that indicates an actually significant synergism again. Note that, in this case, the dose level changes of about 30 % between the two combination conditions.

The third example is the second combination arm of the *Experiment e*. The PTGC of the *e*_2_ arm is reported in Fig. 6, right panel, with the experimental data. The experimental data are well described by the model (RMSE = 0.08 g). In this case, the antagonistic combination index *AC* = 14 % predicted by the model suggests a moderate antagonism between the two drugs, confirmed by the real value computed by fitting the TGI model against the second combination arm data (*AC* = 9 %). In this case, the dose level changes of about 100 % between the to combination conditions.

## Conclusion

Considering the great interest and the relevant effort put in the design and assessment of new anticancer combination therapies that represent the actual strategy widely adopted in clinical oncology, we proposed a new model able to characterize and quantify the interaction of co-administered drugs and to get guidance for early discovery and development. Indeed, even if several approaches have been proposed in literature, none of them provides a generally valid tool for the assessment of the drug effect interaction nature and intensity in combination administration studies in xenograft mice. For example, the applicability field of the model proposed by Koch et al. [14] is restricted only to drugs that have the same mechanism of action, in terms of tumor cell death rate. In fact, in the *Simeoni TGI model* (from which this model derives), the parameter *k*_1_ (the tumor cell death rate) is known to be linked to the drug mechanism of action. Substantially, different *k*_1_ values are supposed to be related to different mechanisms of action. The *Koch model* includes only one *k*_1_ parameter, implicitly assuming that both drug have to be characterized by the same tumor cell death rate. If the two drugs (when administered as single agent) show a different *k*_1_ values, this combination model is not able to take into account it. Moreover, in *Koch model*, since only one drug potency is modulated through the interaction term, a-priori strong assumptions on the interaction effect are required. The first restriction also characterizes the two slightly different models proposed by Goteti et al. [10]. Both models lack of general validity: in the first one, the drug interaction term is assumed to be exactly equal to the product of the activities of the two drugs when given as single agent; in the second one, the combination term also includes a delay between the action of a drug and its concentration level, neglecting one of the single drug activities.

The TGI model here proposed wants to be an answer and a valid way to definitely fill a well acknowledged gap. From a modeling point of view, an interaction term γ has been introduced only on the proliferating cell compartment of a two drugs TGI model, in which a 4 × 4 mortality matrix allows to take into account for different mechanisms of action. The effect of drugs interaction on non-proliferating cells has been neglected since it has a very limited impact on the total tumor weight, and then, it is not appreciable on the real data. The proposed model is simple enough to be identified on the experimental data available during the preclinical phase, in which many drug combinations are tested, but each drug combination might be tested only at one or two dose levels. Gaining immediately information on the compounds under study about their possible future use in combination therapy may substantially impact on the subsequent phases of the drug development process.

The estimation of the interaction term allows an easy evaluation of the nature of the interaction: positive or negative γ values indicate a possible synergistic or antagonistic nature of the drug effect interaction, respectively. In order to provide an understandable measure of the strength of the interaction, two additional indexes (called *synergistic/antagonistic combination index*) were defined starting from a largely used antitumor efficacy index (TEI) originally introduced in [18, 26] and here evaluated for a combination treatment.

Note that, as in the *Simeoni TGI model*, the TGI combination model assumes a linear relationship between drug concentrations and the killing rate. However, even if in all the analyzed experiments a linear relationship adequately described the data, our experience with single drug administrations suggests that, in few cases, it is possible to have nonlinear drug effects, e.g., saturable effects, mainly when a wide range of concentration is tested. Following the general approach here proposed, the nonlinear drug effects should be learnt in single drug administrations and then embedded in the combination model. These cases can be easily managed, modifying the *k*_2_ *c*(*t*) term following the discussion reported in [17, 19] in which a more general TGI model framework is presented.

The strongest and most important point of the model here proposed is that it is able to characterize the drug potency and the pharmacological drug interaction independently from dose levels and schedules, at least in a reasonable range of experimental conditions as discussed in the "Results" section. Then, it is possible to predict the tumor growth in new combination arms, starting from the interaction term estimated for the same considered compounds given in a different administration schedule/dose. Modeling the time course of anticancer effects of co-administered drugs is desirable, since it may facilitate optimization strategies of combination therapies, thus reducing times and costs. In fact, this approach could represent a guide in the definition of Phase I studies, in the selection of clinical doses and optimal schedules to maximize tumor suppression. Finally, it could be also applied to test pharmacological hypotheses, such as the synergistic inhibition of two complementary signaling pathways.

The relevance and general applicability of the proposed PK-PD model was demonstrated analyzing 11 studies involving three tumor cell lines, four new compounds as well as four drugs already on the market. It has been shown that this approach is of practical use as it can be applied to assess combination therapies in routinely performed xenograft experiments, without requiring drug-specific mechanistic hypothesis. It enables to identify synergistic drug combinations when detailed information on the drug action pathways are not available, filling the lack of a generally applicable combination model. For these reasons, this model can be considered an indispensable tool in the preclinical drug development and a crucial advance in the knowledge as it integrates the previous information (also on single drug administrations) to improve the decision making.

## Electronic supplementary material

Below is the link to the electronic supplementary material.

PDF (163 KB)

PDF (124 KB)

PDF (95 KB)

PDF (116 KB)

## Footnotes

1 Drug C1 is an aurora inhibitor, Drug C2 a polo like kinase (PLK) inhibitor, Drug C4 and Drug C5 are multikinase inhibitors. Note that, Drug C1 and Drug C2 are the same compounds analyzed in [20]. For the sake of readability, we referred to them using the same notation.

2 Drug C1, in the single agent arm, was administered two times per day for nine days starting from day 9.

3 The first administration of Drug C4 was given at day 9 in the single agent arms, whereas was given at day 10 in the combination arms to avoid an overlapping in the administration of the two compounds.

4 This is one of the weighting strategies made available by WinNonlin and Matlab, adopted in several other studies, to speed-up the estimation process in order to approximate an additive proportional error model.

5 This is a common choice available in software tools such as WinNonlin to avoid that the model parameter estimation procedure is more driven by some arms. However, in the cases reported in this paper, results are not strongly affected by the adoption of this strategy, that, therefore, is not crucial in this experimental context.

6 Note that a *CV* ≤ 50 % (under normal distribution hypothesis) implies that the parameter value is statistically distinguishable from zero.

7 The simultaneous estimation of the two combination arms could be also performed with success, if the goal of the analysis was only the characterization of the investigated combination therapy. However, the goal in this section is slightly different: it is the evaluation of the robustness of the proposed approach. Then, the question was to assess how the gamma estimates change from one experiment to another one and to understand what happens if only one of the two experiments was actually performed. This evaluation is a preliminary step to the validation results reported in the “TGI model predictive power” section.
