# Supplementary material for: A predictive pharmacokinetic–pharmacodynamic model of tumor growth kinetics in xenograft mice after administration of anticancer agents given in combination
Source: Cancer Chemother Pharmacol. 2013 Jun 29;72(2):471–82. doi: 10.1007/s00280-013-2208-8 (PMC3718992; doi:10.1007/s00280-013-2208-8)
Supplement: Supplementary file 1 — PDF (163 KB) [file 280_2013_2208_MOESM1_ESM.pdf]

SUPPLEMENTARY FILE S1 - TEI_{comb}

The TEI is defined for a finite treatment as the asymptotic horizontal distance between the treated and untreated growth curves. Following [18] (Definition 12), $TEI \equiv \lim_{w\to+\infty}(\tau_t - \tau_c)$, where, for a given weight w, $\tau_t = \tau_t(w)$ and $\tau_c = \tau_c(w)$, being $\tau_t(w)$ and $\tau_c(w)$ the time in which the tumor growth reaches the weight w in the treated and control groups, respectively.

In order to compute TEI_{comb}, it is necessary to derive $\tau_c(w)$ and $\tau_{comb}(w)$. From [18](*A.5. Proposition 14*), $\tau_c(w) = [w - \tilde{w}]/\lambda_1 + [\ln(\tilde{w}) - \ln(w_0)]/\lambda_0$. From eq.1 of the main text, so long $w(t) \leq \tilde{w}$, we have that

$$\dot{x}_{00}(t) = \lambda_0 x_{00}(t) - (k_{2a}c_a(t) + k_{2b}c_b(t) + \gamma c_a(t)c_b(t))\, x_{00}(t)$$

and integrating and solving respect to t, we obtain:

$$\begin{aligned} t &= \frac{1}{\lambda_0}\left[\ln(x_{00}(t)) - \ln(w_0) + k_{2a}AUC_{c_{a(t)}} + k_{2b}AUC_{c_{b(t)}} + \gamma AUC_{c_{a(t)}c_{b(t)}}\right] \\ &\equiv \tau_{comb}(w) \text{ for } w \leq \tilde{w} \end{aligned}$$

where $AUC_{c_{a(t)}} = \int_0^t c_a(t)dt$, $AUC_{c_{b(t)}} = \int_0^t c_b(t)dt$, $AUC_{c_{a(t)}c_{b(t)}} = \int_0^t c_a(t)c_b(t)dt$.

For a given w, define $\epsilon_1(w) = w - x_{00}(\tau_{comb}(w))$, i.e. the weight of non-proliferating cells when the total tumor weight is equal to w. Note that, for a finite duration treatment, $\lim_{t\to+\infty}\epsilon_1(w) = 0$. Under the further hypothesis that the treatment end before the tumor reaches $\tilde{w}$ (i.e, $c_a(t) = 0$ and $c_b(t) = 0$ for $t \geq \tilde{\tau}_{comb} \equiv \tau_{comb}(\tilde{w})$ becomes:

$$\tilde{\tau}_{comb} = \frac{1}{\lambda_0}\left[\ln(\tilde{w} - \epsilon_1(\tilde{w})) - \ln(w_0) + k_{2a}AUC_{c_a} + k_{2b}AUC_{c_b} + \gamma AUC_{c_a c_b}\right]$$

For $t > \tilde{\tau}_{comb}$, being $w(t) > \tilde{w}$, we have that

$$\dot{x}_{00}(t) = \lambda_1 \frac{x_{00}(t)}{w(t)} < \lambda_1$$

or equivalently

$$x_{00}(t) = x_{00}(\tilde{\tau}_{comb}) + \lambda_1(t - \tilde{\tau}_{comb}) - \epsilon_2(t)$$

with $\epsilon_2(t) > 0$ and $\lim_{t\to+\infty}\epsilon_2(t) = \bar{\epsilon}_2 > 0$. Therefore,

$$t = \frac{1}{\lambda_1}\left[w - \epsilon_1(w) - (\tilde{w} - \epsilon_1(\tilde{w})) + \epsilon_2(\tau(w))\right] + \tilde{\tau}_{comb} \equiv \tau_{comb}(w) \text{ for } w \geq \tilde{w} \quad (8)$$

from which being $\lim_{t\to+\infty}\epsilon_1(w) = 0$ and $\lim_{t\to+\infty}\epsilon_2(t) = \bar{\epsilon}_2 > 0$ follows

$$\begin{aligned} TEI_{comb} &= \lim_{w\to+\infty}(\tau_{comb} - \tau_c) \\ &= \frac{1}{\lambda_0}\left[\ln(\tilde{w} - \epsilon_1(\tilde{w})) - \ln(\tilde{w}) + k_{2a}AUC_{c_a(t)} + k_{2b}AUC_{c_b(t)} + \gamma AUC_{c_a c_b}\right] + \\ &+ \frac{1}{\lambda_1}\left[\epsilon_1(\tilde{w}) + \bar{\epsilon}_2\right] \end{aligned}$$

Moreover, if $\epsilon_1(\tilde{w}) \simeq 0$, i.e. $x_{00} \simeq w$ when the tumor growth switches from the exponential growth phase to the linear one, a first order Taylor expansion of the logarithm yields:

$$TEI_{comb} \simeq \frac{1}{\lambda_0}\left[k_{2a}AUC_{c_a} + k_{2b}AUC_{c_b} + \gamma AUC_{c_a c_b} - \epsilon_1(\tilde{w})/\tilde{w}\right] + \frac{\bar{\epsilon}_2}{\lambda_1}$$

Finally, observing that $x_{00}(\tilde{\tau}_{comb}) \simeq w(\tilde{\tau}_{comb})$ implies $\frac{\bar{\epsilon}_2}{\lambda_1} \simeq 0$, the following final approximation is obtained.

$$TEI_{comb} \simeq \frac{k_{2a}AUC_{c_a} + k_{2b}AUC_{c_b} + \gamma AUC_{c_a c_b}}{\lambda_0} \tag{9}$$
