# Supplementary material for: A predictive pharmacokinetic–pharmacodynamic model of tumor growth kinetics in xenograft mice after administration of anticancer agents given in combination
Source: Cancer Chemother Pharmacol. 2013 Jun 29;72(2):471–82. doi: 10.1007/s00280-013-2208-8 (PMC3718992; doi:10.1007/s00280-013-2208-8)
Supplement: Supplementary file 2 — PDF (124 KB) [file 280_2013_2208_MOESM2_ESM.pdf]

SUPPLEMENTARY FILE S2 - The index $\varDelta$

The evaluation of the horizontal distance between the PTGC (obtained by the zero-interaction model) and that obtained by the new model in combination regimen is a very interesting measure of the additional amount due to the drug-drug interaction.

As previously, let us consider the perturbed-growth curve in combination regimen under the assumption of no-interaction between drugs effects in order to compute $\tau_{add}(w)$. Observing that $\tau_{add}(w)$ can be obtained from $\tau_{comb}(w)$ imposing $\gamma = 0$ and substituting $\epsilon_1 \mapsto \epsilon_{1add}$ and $\epsilon_2 \mapsto \epsilon_{2add}$ in 8, we have

$$\begin{aligned}\tau_{add}(w) &= \frac{1}{\lambda_1}\left[w - \epsilon_{1add}(w) - (\tilde{w} - \epsilon_{1add}(\tilde{w})) + \epsilon_{2add}(w)\right] \\ &+ \frac{1}{\lambda_0}\left[\ln(\tilde{w} - \epsilon_{1add}(\tilde{w})) - \ln(w_0) + k_{2a}AUC_{c_a} + k_{2b}AUC_{c_b}\right]\end{aligned}$$

Being by definition

$$\begin{aligned}\varDelta &= \lim_{w\to+\infty}(\tau_{comb} - \tau_{add}) \\ &= \frac{1}{\lambda_1}\left[\epsilon_1(\tilde{w}) - \epsilon_{1add}(\tilde{w}) + \bar{\epsilon}_2 - \bar{\epsilon}_{2add}\right] + \\ &+ \frac{1}{\lambda_0}\left[\ln(\tilde{w} - \epsilon_1(\tilde{w})) - \ln(\tilde{w} - \epsilon_{1add}(\tilde{w})) + \gamma AUC_{c_a c_b}\right]\end{aligned}$$

If also $\epsilon_{1add}(\tilde{w}) \simeq 0$, a first order Taylor expansion of the logarithm yields:

$$\varDelta \simeq \frac{\bar{\epsilon}_2 - \bar{\epsilon}_{2add} + \epsilon_1(\tilde{w}) - \epsilon_{1add}(\tilde{w})}{\lambda_1} + \frac{1}{\lambda_0}\left[\gamma AUC_{c_a c_b} - \epsilon_1(\tilde{w})/\tilde{w} + \epsilon_{1add}(\tilde{w})/\tilde{w}\right]$$

observing that $x_{00}(\tilde{\tau}_{add}) \simeq w(\tilde{\tau}_{add})$ implies $\frac{\bar{\epsilon}_{2add}}{\lambda_1} \simeq 0$, the final approximation is

$$\varDelta \simeq \frac{\gamma AUC_{c_a c_b}}{\lambda_0} \tag{10}$$
