# Supplementary material for: A predictive pharmacokinetic–pharmacodynamic model of tumor growth kinetics in xenograft mice after administration of anticancer agents given in combination
Source: Cancer Chemother Pharmacol. 2013 Jun 29;72(2):471–82. doi: 10.1007/s00280-013-2208-8 (PMC3718992; doi:10.1007/s00280-013-2208-8)
Supplement: Supplementary file 3 — PDF (95 KB) [file 280_2013_2208_MOESM3_ESM.pdf]

SUPPLEMENTARY TABLE 1

Table 1 PK parameters of anticancer drugs.

Drug	V_1 [l/kg]	K_{10} [1/day]	K_{12} [1/day]	K_{21} [1/day]	K_{01} [1/day]
$5-fluorouracil$	0.714	151	5.62	2.30	.
$Cisplatin$	0.395	106	109	47.3	.
$Irinotecan$	4.85	13.3	0.275	1.48	.
$Gemcytabine$	0.887	97.3	20.4	45.2	.
$Drug\ C1$	1.420	28.1	4.94	5.58	.
$Drug\ C2$	2.13	49.2	141	10.4	18.8
$Drug\ C4$	13.3	3.36	.	.	206
$Drug\ C5$	0.577	89.0	13.9	13.7	.
