# Supplementary material for: A predictive pharmacokinetic–pharmacodynamic model of tumor growth kinetics in xenograft mice after administration of anticancer agents given in combination
Source: Cancer Chemother Pharmacol. 2013 Jun 29;72(2):471–82. doi: 10.1007/s00280-013-2208-8 (PMC3718992; doi:10.1007/s00280-013-2208-8)
Supplement: Supplementary file 4 — PDF (116 KB) [file 280_2013_2208_MOESM4_ESM.pdf]

SUPPLEMENTARY TABLE 2

Table 2 PD parameters estimated from control and single agent arms. Percentage coefficients of variation are reported (between brackets) only for the parameters estimated as part of this work, the other ones were estimated in [20]

	Experiment a	*Experiment b*	*Experiment c*	*Experiment d*	*Experiment e*	*Experiment f*
Drug A	Drug C1	Drug C1	Drug C2	Drug C2	Drug C4	Drug C5
Drug B	Gemcytabine	Cisplatin	CPT-11	5-FU	Gemcytabine	CPT-11
λ_0 $[day^{-1}]$	0.149	0.326<br>(7%)	0.0880	0.0903	0.153<br>(4%)	0.231<br>(7%)
λ_1 $[g\ day^{-1}]$	0.203	0.286<br>(8%)	0.472	0.0459	0.223<br>(11%)	0.252<br>(13%)
k_{1_A} $[day^{-1}]$	2.24	0.608<br>(15%)	2.63	2.46	62.6<br>(> 100%)	1.30<br>(31%)
k_{2_A} $[\mu\ M^{-1}\ day^{-1}]$	0.0512	0.186<br>(9%)	0.232	0.241	0.0526<br>(6%)	0.292<br>(10%)
k_{1_B} $[day^{-1}]$	1.68	0.155<br>(> 100%)	0.490	0.612	1.49<br>(58%)	0.109<br>(36%)
k_{2_B} $[\mu\ M^{-1}\ day^{-1}]$	0.0984	1.89<br>(48%)	0.408	0.0837	0.104<br>(10%)	1.65<br>(9%)
w_0 $[g]$	0.0566	0.0211<br>(19%)	0.0891	0.0884	0.0533<br>(9%)	0.0361<br>(15%)
